# Obesity and temporomandibular joint disorders: a systematic review and meta-analysis

**DOI:** 10.1186/s12903-023-03322-2

**Published:** 2023-08-29

**Authors:** Xia Wang, Yan Yang, Linni Lin, Qianqian Yao, Jingjing Zhang

**Affiliations:** 1 Center of Stomatology, The Second Xiangya Hospital of Central South University, 139 Renmin Middle Road, Changsha, Hunan 410011 China; 2 National Clinical Research Center for Metabolic Diseases, Metabolic Syndrome Research Center, Key Laboratory of Diabetes Immunology (Central South University), Ministry of Education, and Department of Metabolism and Endocrinology, The Second Xiangya Hospital of Central South University, Changsha, 410011 Hunan China

**Keywords:** Obesity, Fat body, Body mass index, Temporomandibular joint disorders, Mouth diseases

## Abstract

**Background:**

Temporomandibular joint disorders (TMD) is the most common non-dental pain complaint in the maxillofacial region, which presents a variety of symptoms and signs, including temporomandibular joints (TMJ) and masticatory muscle pain, joint noise, tinnitus, headaches, irregular or restricted mandibular function, masticatory difficulty, and restricted mouth opening. When comes to the relationship between obesity and TMD, it has remained controversial and inconsistent, therefore, we first conducted this meta-analysis to estimate the unclear relationship between obesity and TMD.

**Methods:**

Searches were conducted in PubMed, Web of Science, Embase, and Cochrane Library. Subjects were divided into five groups according to BMI level in this study, including the normal weight group: 18.5 ≤ BMI < 25, overweight group: 25 ≤ BMI < 30, obesity group: BMI ≥ 30, control group: BMI < 25, and overweight and obesity group: BMI ≥ 25. Statistics analyses were conducted using Stata (15.0). The number of PROSPERO was CRD42022368315.

**Results:**

Eight studies were included in this study, and six articles with a total of 74,056 participants were synthesized for meta-analysis. Compared to normal weight individuals, overweight and obesity together decreased the risk of TMD (OR = 0.66, 95% CI = 0.46–0.95), and it was significantly decreased by obesity alone (OR = 0.58). Moreover, it was lower in obesity compared with control subjects (OR = 0.83, 95% CI = 0.73–0.94). Furthermore, in overweight and obese individuals, it was much lower in obesity than in overweight (OR = 0.82, 95% CI = 0.71–0.94).

**Conclusions:**

Obesity is not a risk factor for TMD, and maybe a protective factor for TMD, of which patients with larger BMI are less likely to suffer from TMD pain. Therefore, the value of BMI should be taken into consideration in the assessment of TMD.

**Supplementary Information:**

The online version contains supplementary material available at 10.1186/s12903-023-03322-2.

## Background

The most widelyaccepted definition of temporomandibular joint disorders (TMD) is “a group of musculoskeletal and neuromuscular conditions that involve the temporomandibular joints(TMJ), the masticatory muscles, and all associated tissues’’ from The American Association for Dental Research [1]. It presents a variety of symptoms and signs, including TMJ and masticatory muscle pain, joint noise, tinnitus [2], headaches, irregular or restricted mandibular function, masticatory difficulty, and restricted mouth opening [3]. Moreover, there also have a interaction between TMD and neck pain in primary headache patients [4], and which is the most common non-dental pain complaint in the maxillofacial region, and approximately 31% of adults, and 11% of children, would experience the pain in their lifetime [5]. In children and adolescence, the female had a higher prevalence of TMD compared to male [6]. Yet, there was no difference in TMD prevalence between pregnant and non-pregnant women in childbearing age [7]. Furthermore, the original Research Diagnostic Criteria for Temporomandibular Disorders (RDC/TMD) was diagnosed using the new Axis II protocol, which retains augmented RDC/TMD screening instruments to assess jaw function as well as behavioral and additional psychosocial factors [8]. And radial Extracorporeal Shock Wave Therapy (rESWT) combined with physical therapy could be an effective therapy to relieve pain and improve function in muscle-related TMD patients [9].

The etiology of TMD is usually multi-factorial, and the exact cause of symptoms may be difficult to determine. Which could be divided into both physical and psychosocial factors [10–12]. Arthrogenous, and the more common myogenous origins can be broadly attributed to the physical causes, which are characterized as disruption of the internal aspect of the joint, and usually pertains to an articular disc that has been displaced, such as abnormal occlusion, mandibular asymmetry [12], bruxism, teeth grinding, lip biting, joint capsule inflammation or oxidative damage [13], muscle spasm, and abnormalities in the intraarticular disk [10, 14]. The severity for internal derangements of TMJ was classified as early stage, early/intermediate stage, intermediate stage, intermediate/late stage, and late stage, according to clinical, radiologic, and surgical findings [15]. Moreover, apart from physical causes, the association between biopsychosocial factors and TMD was also be researched by many studies, and symptoms of TMD can be exacerbated by depression, anxiety, and stress [16–18].

However, when comes to the relationship between obesity and TMD, it has remained controversial and inconsistent. One article demonstrated that obesity did not show any association with TMD pain in adolescents [19]. Another previous study showed that women with TMD had lower BMI, waist circumference, metabolic syndrome, and prevalence of diabetes, nevertheless, men with TMD did not show any statistical significant differences [20]. In a single regression analysis, TMD pain was obviously increased with the percentage of total body fat, whereas the initial association disappeared in multivariable analysis [21]. Therefore, the purpose of this meta-analysis is to summarize all the studies so far to explore the unclear relationship between obesity and TMD in females and males. As far as we know, this is the first systematic review and meta-analysis that evaluates the association between the different values of body mass index (BMI) and the risk of TMD.

## Methods

The methods of this study were performed in accordance with the Preferred Reporting Items for Systematic Reviews and Meta-analyses (PRISMA) statement [22] (PROSPERO No. CRD42022368315). According to the principle of PICO (Patient, Intervention, Comparison, and Outcome), the focused question was addressed: in patients, different BMI index, have a different rate of TMD?

### Eligibility criteria

The inclusion criteria containing the study were as follows: Participants: (1) Total population should be greater than 20; (2) The number of patients with TMD in the group of obesity and normal weight should be recorded accurately; (3) No gender preference and both males and females were included; Intervention: (1)Obesity and large BMI index; Comparison: (1) Different BMI index; Outcome: (1) The rate of TMD in groups based on different BMI index; Study: (1) Randomized controlled trials, cohort studies, and case–control studies were included.

The exclusion criteria were as follows: (1) The number of subjects was less than 20; (2) The included research object only included men or women; (3) The rate of TMD in obese and normal weight group cannot be obtained simultaneously; (4) The type of article was designed as review, meta-analysis, and case report.

The PICOS principles included in the literature in this meta-analysis were shown in Table 1.

**Table 1:** PICOS criteria for included studies

Parameter	Inclusion criteria
P	Total participants > 20, accurately recorded the number of patients with TMD in the group of obesity and normal weight, no gender preference (both males and females include),
I	Obesity and large BMI index
C	Different BMI index
O	The rate of TMD in groups based on different BMI index
S	Randomized controlled trials, cohort studies, and case–control studies

*P* Participants

*I* Intervention

*C* Comparator

*O* Outcomes

*S* Study Design

### Information source and search strategy

The four electronic bibliographic databases (PubMed, Web of Science, Embase, and Cochrane Library) were searched, with no time or language restrictions, up to October 26, 2022. (The complete search strategy for each database was attached in the supplementary material).

### Study selection

After removing duplicate articles, the titles and abstracts of the searched studies were beginning to screen by two independent reviewers (XW and YY). Articles that meet the inclusion criteria in the first analysis, as well as titles and abstracts that did not give sufficient information to be judged, were further evaluated in full text, while those that did not meet the inclusion criteria were excluded. Then, the remaining literature was strictly screened according to the inclusion and exclusion criteria by scanning the full text of the article. The contradiction between the two reviewers in the evaluation of whether an article was included or excluded was resolved by consultation with a third examiner (QY).

### Quality assessment

The Newcastle–Ottawa scale (NOS) [23] was used to assess the quality of observational studies and non-randomized control trials, which included three categories with a maximum of nine stars, as follows: four stars for selection, two stars for comparability, and three stars for results. The two reviewers (XW and YY) independently used the NOS [23] to assess the studies that met the inclusion criteria. Inconsistency in the quality evaluation of studies was solved by a discussion with a third researcher (QY). Studies with NOS scores of more than 6 were defined as high quality.

### Data extraction

The following data were extracted from the article by one of the reviewers (XW) using the standardized data abstraction table: the first author, year, country, the type of study design, the means of TMD evaluation, the number of patients, mean age of participants, sex ratio, BMI of each group, odds ratio (OR) of TMD.

### Data analysis and synthesis

The software of Stata (15.0) was used to conduct statistical analyses. *P* < 0.05 was considered statistically significant. Data of patients with TMD from the included studies were collected to calculate OR with a 95% confidence interval (95% CIs), respectively. Heterogeneity among the included studies was assessed by using I‐square (I^2^) and Cochran’s *Q* test. I^2^ values range from 0 to 100%, if I^2^ values below 25% are considered as low, 25 to 50% as moderate, and above 75% as high heterogeneity [24]. When *P* < 0.05 and/ or I^2^ > 50%, random-effect models were adopted to evaluate the pooled data, while when *P* > 0.05 and/ or I^2^< 50%, fixed-effect models were adopted. Publication bias was assessed by the funnel plot, Egger’s tests, and the trim-and-fill method [25]. Subgroup analysis was conducted based on BMI. The classification of subjects into five groups according to BMI, which included the normal weight group: 18.5 ≤ BMI < 23, obesity I group: 25 ≤ BMI < 30, obesity II group: BMI ≥ 30, non-obesity group: BMI < 25, and obesity group: BMI ≥ 25. Similarly, sensitivity analysis was conducted to explore whether the meta-analysis results could be affected markedly by a particular study.

## Results

### Study selection

**Fig. 1:**
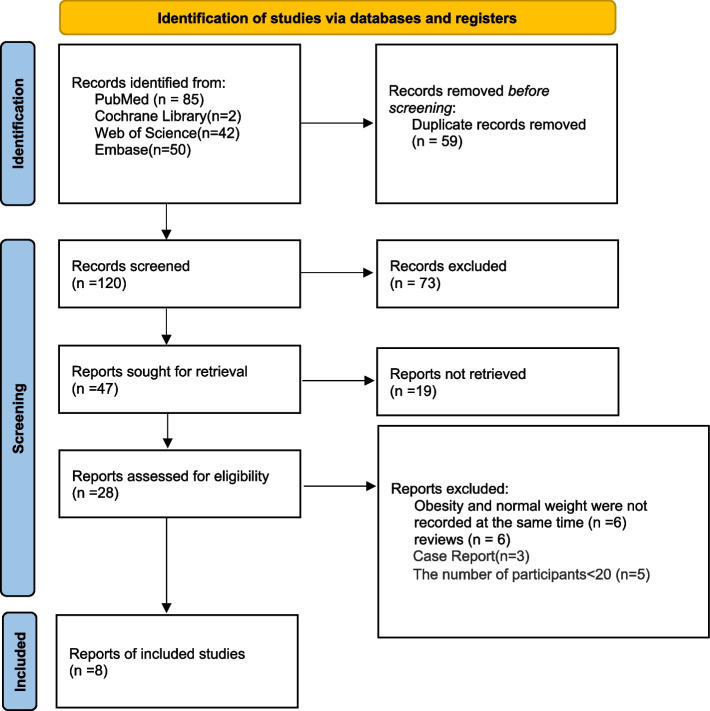
PRISMA flow diagram of the study selection process

The flow diagram of the selection process of included studies was shown in Fig. 1. A total of 179 papers were yielded: 85 articles from PubMed, 2 from Cochrane Library, 42 from Web of Science, and 50 from Embase, from which 120 articles remained after the elimination of duplicate studies. After the screening of titles and abstracts, 73 studies were excluded, and 47 articles were found to be eligible for full-text assessment. A total of 20 papers were excluded as they did not conform to the inclusion criteria:

The number of patients included in the study in 5 papers was less than 20; In 6 literature the obesity and normal weight group were not recorded at the same time; Reviews and case report in 9 papers.

Finally, 8 articles were included in our systematic review [19, 20, 26–31],and 6 articles were combined for meta-analysis [19, 20, 26, 27, 29, 30]. Two articles were not included in the meta-analysis due to the different diagnosis methods of TMD, which were reported by themselves through the questionnaire [28, 31].

### Study and patient characteristics

Detailed characteristics of the 8 included studies were presented in Table 2. Four articles were designed as cohort [26, 27, 29, 30], and four studies were designed as cross-sectional type [19, 20, 28, 31]. All included studies were published between 2013 [30] and 2021 [26, 28, 29]. Four articles were conducted by Korea [20, 26, 27, 29], one in Brazil [19], Finland [28], USA [30], and Turkey [31] respectively.

**Table 2:** Description of the characteristics of eligible studies reporting the association between obesity and TMD

Study Characteristics				TMD evaluation	Population Characteristics				Case (n)	OR	95%CI
Author	**Year**	**Country**	**Study Design**		**Subjects** **(n)**	**Age**	**Gender** **(Male/Female)**	**BMI(n)**			
So Young Kim (1)	2021	Korea	Cohort	Clinical examinations	9040	NR	4245/ 4795	Underweight: < 18.5 (215) Normal: ≥ 18.5 to < 23 (3625) Overweight: ≥ 23 to < 25 (2645) Obese I: ≥ 25 to < 30 (2370) Obese II: ≥ 30 (185)	43 725 529 474 37	NR	NR
So Young Kim (2)	2021	Korea	Cohort	Clinical examinations	8105	NR	3685/ 4420	Underweight: < 18.5 (190) Normal: ≥ 18.5 to < 23 (3185) Overweight: ≥ 23 to < 25 (2360) Obese I: ≥ 25 to < 30 (2225) Obese II: ≥ 30 (145)	38 637 472 445 29	NR	NR
Soo-Hwan Byun	2020	Korea	Cohort	Clinical examinations	19,420	NR	8765/ 10,655	Underweight: < 18.5 (497) Normal: ≥ 18.5 to < 23 (7131) Overweight: ≥ 23 to < 25 (5275) Obese I: ≥ 25 to < 30 (5941) Obese II: ≥ 30 (576)	112 1530 1104 1056 82	NR	NR
Ossi Miettinen	2021	Finland	cross-sectional	Self-reported facial pain and symptoms	8677	19.6	8530/ 147	Normal: < 25 (6568) Overweight: ≥ 25 (2109)	NR	1.33	(1.05, 1.68)
Eunmi Rhim	2016	Korea	cross-sectional	Clinical examinations	11,922	NR	5120/ 6802	Normal: < 23 (5500) Overweight: ≥ 23 to < 25 (2707) Obese: ≥ 25 (3715)	477 147 187	NR	NR
Hyo-Geun Choi	2021	Korea	Cohort	Clinical examinations	22,275	NR	9515/ 12,760	Underweight: < 18.5 (599) Normal: ≥ 18.5 to < 23 (7979) Overweight: ≥ 23 to < 25 (6088) Obese I: ≥ 25 to < 30 (6929) Obese II: ≥ 30 (680)	130 1731 1280 1219 95	NR	NR
A.E. Sanders	2013	USA	Cohort	Clinical examinations	2604	NR	1057/ 1547	Underweight/normal: < 25 (1409) Overweight: > 25 to < 30 (712) Obese: ≥ 30 (483)	NR	0.87 1.68	(0.64, 1.20) (1.24, 2.27)
Paula C. Jordani	2019	Brazil	cross-sectional	Clinical examinations	690	12.7 ± 0.76	301/ 389	Underweight: (14) Healthy: (407) Overweight: (159) Obese: (110)	14 407 159 110	0.83 0.98 0.85	(0.18, 3.80) (0.59, 1.60) (0.47, 1.53)
Ahmet Karaman	2021	Turkey	cross-sectional	Diagnosis Criteria Symptom Questionnaire	1528	NR	476/ 1052	Healthy: (600) Overweight: (480) Obese: (448)	252 144 252	NR	NR

*Abbreviations*: Case, subjects with TMD; *NR* Not reported, *TMD* Temporomandibular disorders, *BMI* Body mass index (kg/m^2^)

Patient characteristics for each study were also shown in Table 2. The total number of patients included was 74,056. Three articles divided patients into five groups (underweight, normal, overweight, Obese I, and Obese II) according to BMI (< 18.5, ≥ 18.5 to < 23, ≥ 23 to < 25, ≥ 25 to < 30, ≥ 30) [26, 27, 29]. Two studies were not recorded accurately the value of BMI in each group [19, 31].

### Risk of bias in studies

The quality assessment of the results of the included studies was summarized in Table 3. It was shown that the average score for the quality of the included studies was ≥ 6, except for one article is five [31], indicating most included studies were high-quality.

**Table 3:** Quality assessment of eight studies included in the qualitative evaluation according to the NOS

Author, year, country	Selection				Comparability		Outcome			Score
	**Representativeness of the exposed cohort**	**Selection of the non-exposed control**	**Ascertainment of exposure**	**Outcome of interest no present at start**	**Main factor**	**Additional factor**	**Assessment of outcome**	**Follow-up long enough**	**Adequacy of follow-up**	
So Young Kim, 2021, Korea [26]	★	★	★	0	★	★	★	★	★	8/9
Soo-Hwan Byun, 2020, Korea [27]	★	★	★	0	★	★	★	★	★	8/9
Ossi Miettinen, 2021, Finland [28]	★	★	★	0	★	0	0	★	★	6/9
Eunmi Rhim, 2016, Korea [20]	★	★	★	0	★	★	★	★	★	8/9
Hyo-Geun Choi, 2021, Korea [29]	★	★	★	0	★	★	★	★	★	8/9
A.E. Sanders, 2013, USA [30]	★	★	★	0	★	★	★	★	★	8/9
Paula C. Jordani, 2019, Brazil [19]	0	★	★	0	★	★	★	★	★	7/9
Ahmet Karaman, 2021, Turkey [31]	0	★	★	0	★	0	0	★	★	5/9

### Results of the meta-analysis

The overall effect was divided into four subgroups according to BMI.

#### Overweight and Obesity vs. normal weight

(BMI ≥ 25) vs. (18.5 ≤ BMI < 25)

**Fig. 2:**
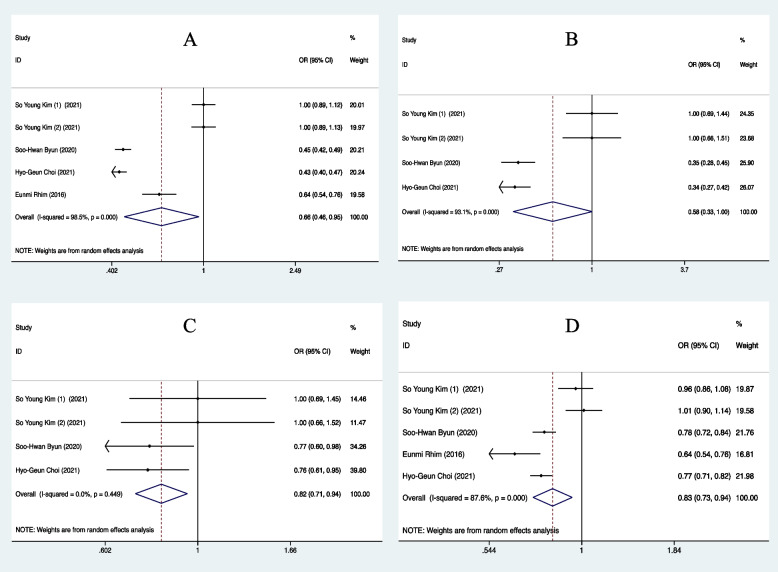
Forest plot of TMD in each subgroup based on different BMI. **A** Overweight and Obesity (BMI ≥ 25) vs. normal weight (18.5 ≤ BMI < 25), **B** Obesity (BMI ≥ 30) vs. normal weight (18.5 ≤ BMI < 25), **C** Obesity (BMI ≥ 30) and overweight (25 ≤ BMI < 30), **D** Overweight and Obesity (BMI ≥ 25) and control (BMI < 25)

**Fig. 3:**
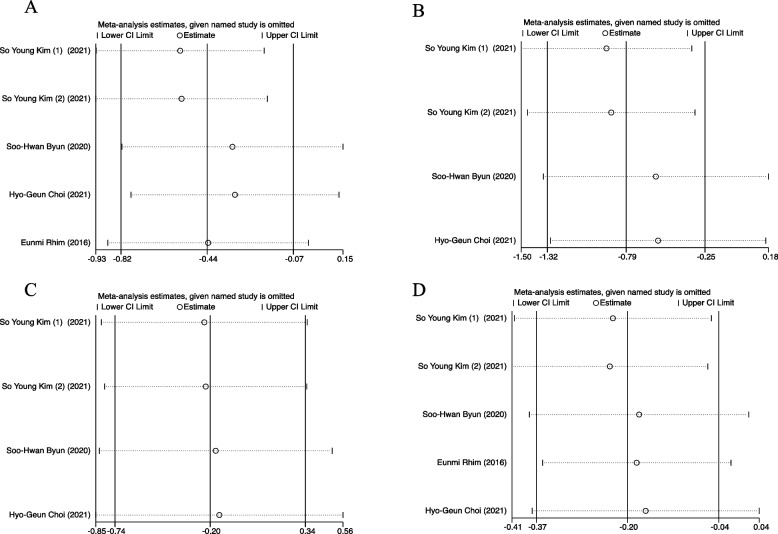
Sensitivity analysis for the effect in each subgroup based on BMI. **A** Overweight and Obesity (BMI ≥ 25) vs. normal weight (18.5 ≤ BMI < 25), **B** Obesity (BMI ≥ 30) vs. normal weight (18.5 ≤ BMI < 25), **C** Obesity (BMI ≥ 30) and overweight (25 ≤ BMI < 30), **D** Overweight and Obesity (BMI ≥ 25) and control (BMI < 25)

A total of 4 papers were included in this subgroup. In this subgroup analysis study, heterogeneity test results revealed significant heterogeneity in these studies (I^2^ = 98.5%, *P* < 0.001) under a random-effects model. The summary result for 5 studies showed that the Overweight and Obesity group significantly decreased the risk of developing into the critical condition of TMD (OR = 0.66, 95%CI = 0.46–0.95) (Fig. 2A). The results of Egger’s test (*p* = 0.153) and inspection of the funnel plots illustrated that there was no publication bias among included studies in the subgroup (Figure S1A) (Table S1). The sensitivity analysis conducted by excluding one study at a time did not change and confirmed the stability of this meta-analysis results (Fig. 3A).

#### Obesity vs. normal weight

(BMI ≥ 30) vs. (18.5 ≤ BMI < 25)

Three papers were categorized in this subgroup. The association between obesity and TMD was strong, with BMI ≥ 30 being around decreased risk to be TMD than normal weight subjects (OR = 0.58) (Fig. 2B). Heterogeneity was very high across studies (I^2^ = 93.1%, *P* < 0.001). A funnel plot was presented in Figure S1B. Visual inspection of the funnel plot and Egger’s test (*p* = 0.017) indicated that publication bias existed (Table S1). However, the trim-and-fill method was further conducted and suggested little evidence of publication bias. The trim-and-fill method estimated that one study was missing due to publication bias and the inclusion of this missing study with imputation produced an effect size similar to the original model (Figure S2A). Moreover, the sensitivity analysis also indicated the stability of our results (Fig. 3B).

#### Obesity vs. overweight

(BMI≥30) vs. (25≤BMI<30)

There were three studies in this subgroup. A random-effects model was used to evaluate TMD between obesity and overweight patients (OR= 0.82, 95% CI=0.71-0.94) (Fig. 2C), which indicated the risk of TMD was much lower in patients with BMI≥30 than those with 25≤BMI<30. Heterogeneity was very low in these included studies (I^2^ = 0.0%, *P* =0.449) (Fig. 2C). The funnel plot for studies in the subgroup was exhibited in Figure S1C. Studies were not distributed relatively symmetrically around the mean, meaning that there was publication bias among included studies. Egger’s test also suggested the occurrence of publication bias (*p* = 0.018) (Table S1). However, little evidence of publication bias was illustrated by using the trim-and-fill method (Figure S2B). Conducting sensitivity analysis further confirmed the stability of the subgroup results (Fig. 3C).

#### Overweight and Obesity vs. control

(BMI≥25) vs. (BMI<25)

Four articles were included in this subgroup. The results of the subgroup for TMD patients were presented in Fig. 2D (OR= 0.83, 95% CI=0.73-0.94), indicating that compared to control individuals (BMI<25), those overweight and obesity patients (BMI≥25) were less likely to suffer from TMD pain. Heterogeneity test results illustrated significant heterogeneity in these included studies of the subgroup (I^2^ = 87.6%, *P* < 0.001). The symmetric funnel plots and the results of Egger’s test (*p* = 0.758) suggested no publication bias presented (Figure S1D) (Table S1). The results of the subgroup were stable by identifying the non-changed results of sensitivity analysis (Fig. 3D).

## Discussion

The relationship between obesity and TMD has been demonstrated in this meta-analysis, surprisingly, obesity is not a risk factor for TMD, and individuals with larger BMI are less likely to suffer from TMD pain. To the best of our knowledge, this is the first meta-analysis to summarize all the studies so far to explore the relationship between obesity and TMD according to the detailed classification of the value of BMI.

Several hypotheses had been proposed and may be the possible explanations for the protective role of obesity in TMD. The first possibility is the chewing ability, which was correlated with dysfunction of the TMD [32], and in the patients with TMD, more changes in chewing function and higher chewing frequency was observed [33]. The bite force, chewing cycle duration and mandibular function was significantly improved after treatment of TMD, indicating that TMD had a negative impact on chewing behavior [34]. Consistent with this, people with a larger BMI had better chewing ability and required less chewing frequency to grind the same amount of food. As a result, people with a larger BMI had a lower risk of TMD compared with people with a smaller BMI.

Bite force can be another explanation for obesity and TMD. The ability of bite force is very important in the masticatory system, which was higher in men aged between 41–50 years, weighing more than 100 kg, and heights between 1.81 and 1.90 m [35]. Bite force was significantly lower in patients with TMD [36, 37], and there was a moderate negative correlation between the two indicators [38]. In TMD patients, not only was a 27% and 6.9% reduction in molar field forces on the second and first molars respectively, but also the center of occlusal forces was located in the most anterior position [39]. Furthermore, TMD individuals had a significantly greater bilateral asymmetry in the occlusal force [40], and the distribution of it on the balancing side was much higher at unilateral TMD [41].

Mental stress could also be another underlying mechanism for the relationship between TMD and BMI. According to a health survey, there was an inverse relationship between perceived stress and obesity among Chinese adults, indicating that people with smaller BMI tend to suffer from more psychological stress [42]. Moreover, stress can promote obesity by reducing physical activity, decreasing sleep duration, and inducing overeating or eating foods with high calories, fat, or sugar [43]. Patients with TMD had much more feelings of distress, anxiety, somatization, and depression [17]. Moreover, diet and/or obesity can directly affect mood, and stress-related mental disorders can also lead to diet habits change [44]. Furthermore, the association between surgery, especially repeated surgery, and psychological disorders were stronger (OR = 2.9) in TMD subjects [45]. Several psychological variables, including reported somatic symptoms, psychosocial stress, and affective distress can predict an increased risk of first-onset TMD [46].

A variety of therapeutic strategies could be implemented for TMD with multi-factorial etiology. The goals of treating TMD include reducing pain and improving mandibular function and it is important to recognize the causes of pain and dysfunction related to TMD for making therapeutic decisions. Conservative ways were usually adopted to treat myogenic TMD, including various medications, such as analgesics, non-steroidal anti-inflammatory drugs (NSAIDs), anxiolytics, and anti-depressants [10], occlusal splints [47], physiotherapy [48], botox injection [49], dry-needling techniques [50], and extracorporeal shock wave therapy [51]. To manage arthrogenic TMD, minimally invasive options containing arthroscopy, arthrocentesis and intra-articular injection, and invasive surgical procedures were commonly performed [52, 53]. In addition, among the patients with symptoms of TMD without obvious physical cause, who have psychosocial comorbidities should be treated by consultation and psychological intervention [10].

### Strengths and limitations of the review

This is, to our knowledge, the first meta-analysis to summarize all the studies so far to explore the relationship between obesity and TMD by classifying the different values of BMI. Rigorous systematic review methods were used, and PRISMA guidelines were followed. Extensive searching was undertaken to identify published and unpublished studies so that all relevant appropriately powered studies were included.

One limitation of this systematic review and meta-analysis is the limited included clinical articles, although all the studies came from very large cohorts or cross-sectional studies, and many people were contained. Moreover, four of the eight included studies were from Korea, which could make the original results from included studies less credible. In addition, results are limited by high study heterogeneity and future research should attempt to better explain the variance of individual studies by analyzing factors such as continuous age, sex ratio, diet habits, and other relevant lifestyles. Therefore, more large-scale clinical studies are required to support or deny our findings in the future.

However, despite these limitations, there was no doubt that the different values of BMI had an impact on the risk of TMD. Obesity may be a protective factor for TMD, of which patients with larger BMI are less likely to suffer from TMD. Up to now, it is still difficult to diagnose TMD in the early stages in patients who experienced masticatory muscle pain without TMJ structural destruction. And our meta-analysis has uncovered a positive relationship between lower BMI and the risk of TMD, indicating that lean people may be the susceptible population to TMD compared with overweight or obese individuals. These results demonstrated that clinical doctors should not miss the susceptibility of patients who only experienced atypical pain in the maxillofacial region, especially lean patients.

## Conclusions

Compared to normal-weight individuals, overweight and obesity (BMI ≥ 25) decreased the risk of TMD, and it was significantly decreased by obesity (BMI ≥ 30). Moreover, it was lower in overweight and obesity (BMI ≥ 25) compared with control subjects (BMI < 25). Furthermore, in overweight and obese individuals, it was much lower in obesity than in overweight. Taken together, the results suggested that obesity is not a risk factor for TMD, and may be a protective factor for TMD, of which patients with larger BMI are less likely to suffer from TMD pain. Therefore, the value of BMI should be taken into consideration in the assessment of TMD. In the future, we could calculate more accurately the risk of TMD in different BMI levels based on numerous studies and to further prevent the occurrence of TMD.

### Supplementary Information

**Additional file 1.**

## Data Availability

All the data used in the meta-analysis presented in this publication is available upon request or contact with the author of Xia Wang.
