# Supplementary material for: Obesity and temporomandibular joint disorders: a systematic review and meta-analysis
Source: BMC Oral Health. 2023 Aug 29;23:607. doi: 10.1186/s12903-023-03322-2 (PMC10466750; doi:10.1186/s12903-023-03322-2)
Supplement: Supplementary file 1 — Additional file 1. [file 12903_2023_3322_MOESM1_ESM.docx]

**Supplementary appendix**

1. **Search Strategies**

An electronic search was performed, with no time or language restrictions, in the following electronic bibliographic databases: PubMed, EMBASE, Web of Science and Cochrane library, up to 0ctober 26, 2022.

**1.1 Web of Science**

1: TS=(obesity) results: 706827

2: TS= (temporomandibular joint disorders) results: 20136

3: #2 AND #1 results: 42

**1.2 PubMed**

**"TMD"[All Fields] AND ("obeses"[All Fields] OR "obesity"[MeSH Terms] OR "obesity"[All Fields] OR "obese"[All Fields] OR "obesities"[All Fields] OR "obesity s"[All Fields])**

**1.3 Embase**

No. Query Results Results

#3. #1 AND #2 50

#2. 'temporomandibular joint disorder' 16,568

#1. 'obesity'/exp 625,432

**1.4 Cochrane library**

ID Search Hits

#1 (obesity) (Word variations have been searched) 53474

#2 ("temporomandibular joint disorder") (Word variations have been searched) 1094

#3 #1 AND #2 2

**Figures**

Figure S1: Funnel plot in each subgroup based on BMI. (A) Overweight and Obesity (BMI≥25) vs. normal weight (18.5≤BMI<25), (B)obesity (BMI≥30) vs. normal weight (18.5≤BMI<25), (C) obesity (BMI≥30) and overweight (25≤BMI<30), (D) Overweight and Obesity (BMI≥25) and control (BMI<25).

A B

**
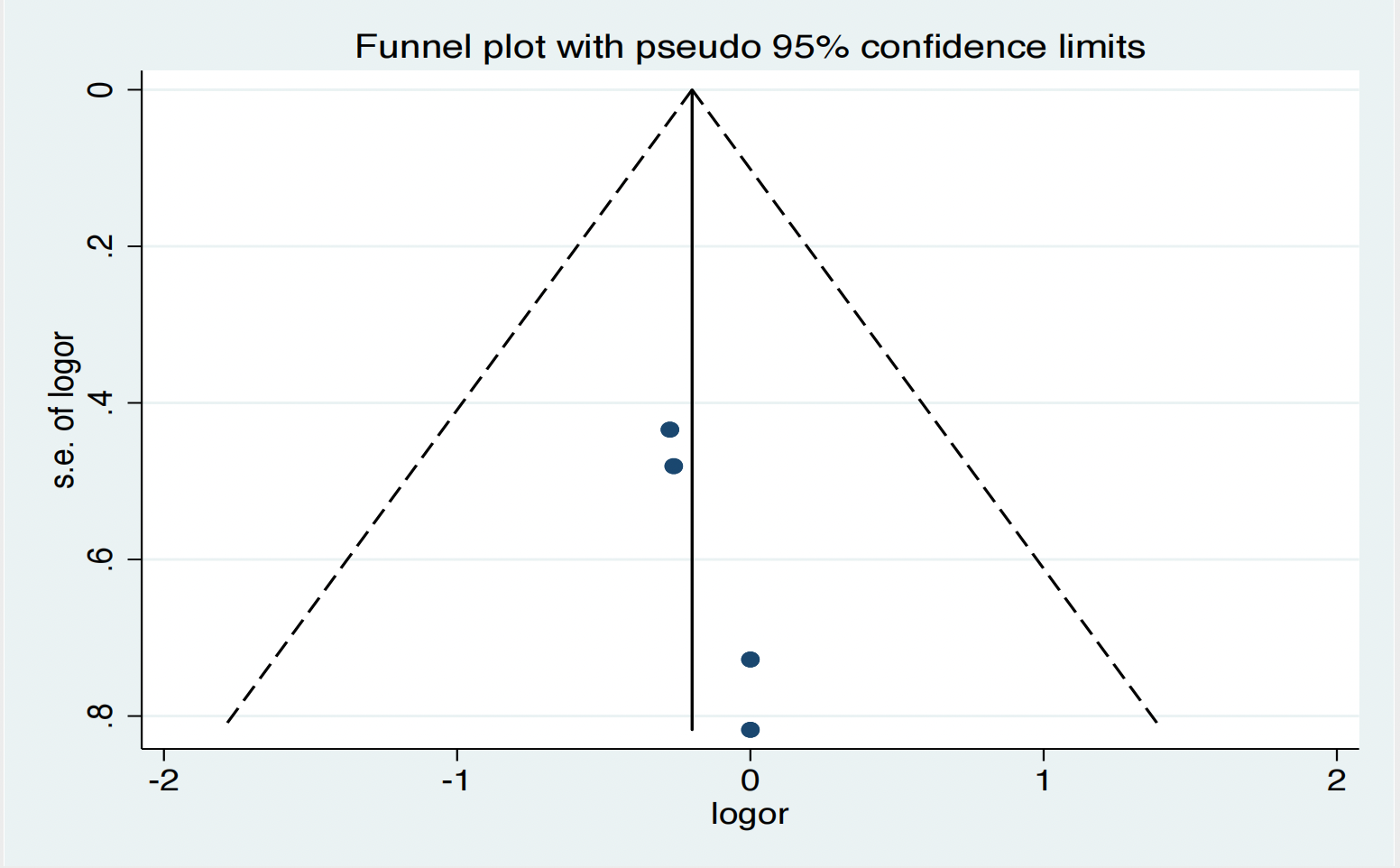
**
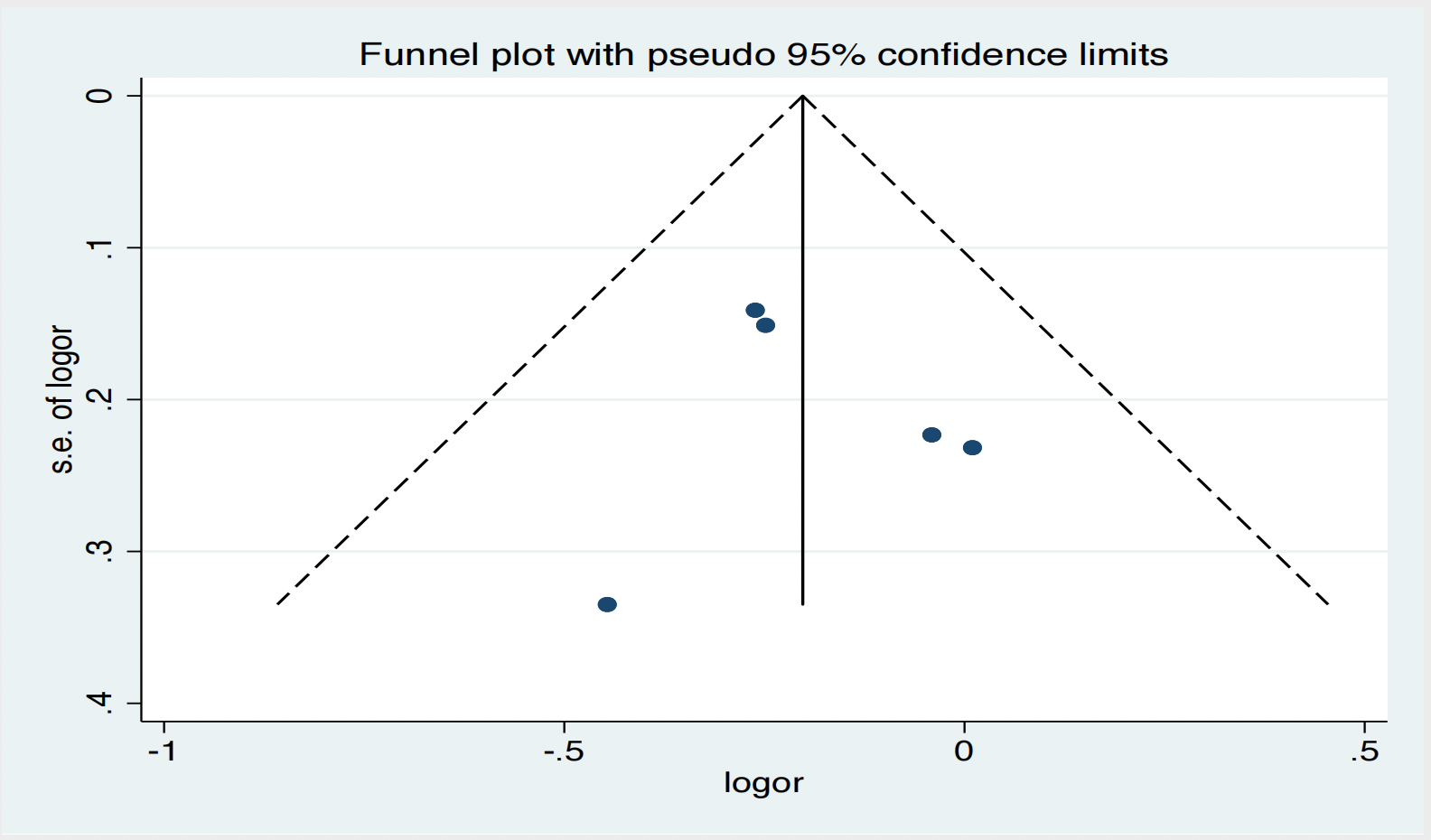


C D

Figure S2: The trim-and-fill method of funnel plot in each subgroup based on BMI. (A) obesity Ⅱ (BMI≥30) vs. normal weight (18.5≤BMI<23), (B) obesity Ⅱ (BMI≥30) and obesity Ⅰ (25≤BMI<30).


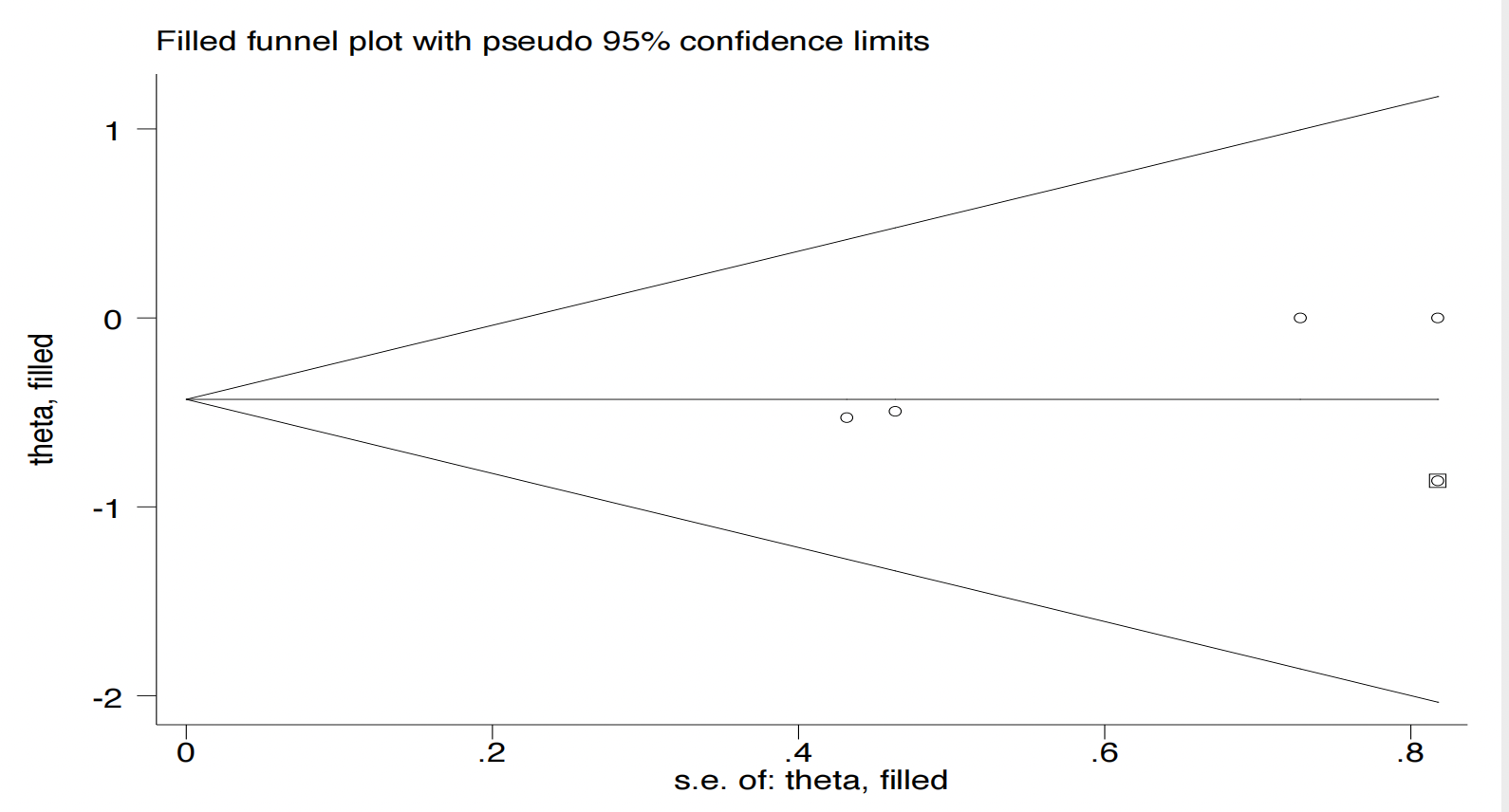

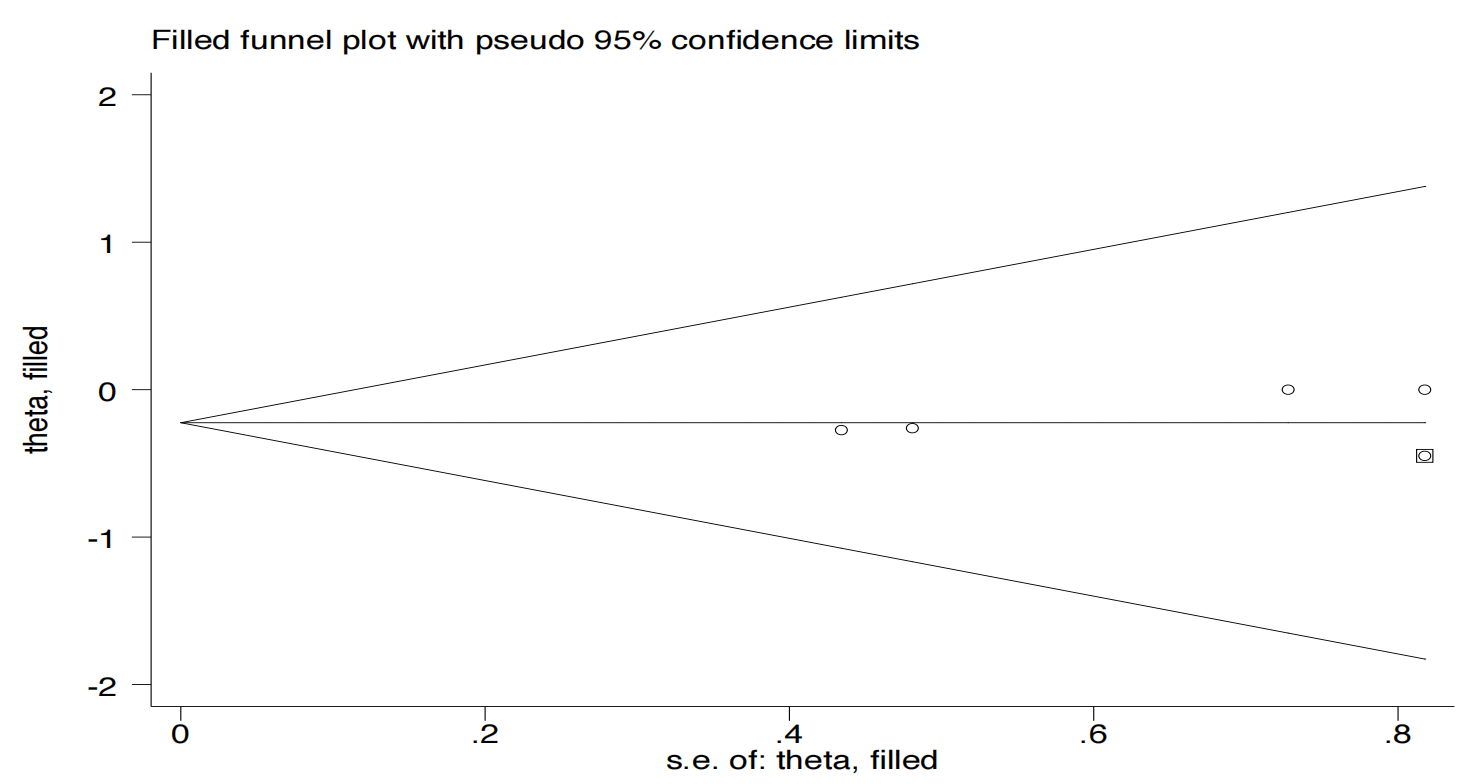


A B

**Tables**

Table S1. The result of Egger’s test for publication bias in each subgroup.

| **Subgroups** | **P value** |
| --- | --- |
|  |  |
| BMI≥25 vs. 18.5≤BMI<25 | P=0.153 |
| BMI≥30 vs. 18.5≤BMI<25 | P=0.017 |
| BMI≥30 vs. 25≤BMI<30 | P=0.018 |
| BMI≥25 vs. BMI<25 | P=0.758 |
